# Primary effusion lymphoma associated with Human Herpes Virus-8 and Epstein Barr virus in an HIV-infected woman from Kampala, Uganda: a case report

**DOI:** 10.1186/1752-1947-5-60

**Published:** 2011-02-14

**Authors:** Lynnette K Tumwine, Rejani Lalitha, Claudio Agostinelli, Simon Luzige, Jackson Orem, Pier Paolo Piccaluga, Lawrence O Osuwat, Stefano A Pileri

**Affiliations:** 1 Department of Pathology, School of Biomedical Sciences, Makerere University College of Health Sciences, Mulago Hill Road, PO Box 7072, Kampala, Uganda; 2 Department of Medicine, School of Medicine, Makerere University College of Health Sciences, Mulago Hill Road, PO Box 7072, Kampala, Uganda; 3 Unit of Hematopathology, Department of Haematology and Oncological Sciences "L and A Seràgnoli", S Orsola- Malpighi Hospital, University of Bologna, Bologna, Italy; 4 Uganda Cancer Institute, PO Box 3935, Kampala, Uganda

## Abstract

**Introduction:**

Primary effusion lymphoma is a recently recognized entity of AIDS related non-Hodgkin lymphomas. Despite Africa being greatly affected by the HIV/AIDS pandemic, an extensive MEDLINE/PubMed search failed to find any report of primary effusion lymphoma in sub-Saharan Africa. To our knowledge this is the first report of primary effusion lymphoma in sub-Saharan Africa. We report the clinical, cytomorphologic and immunohistochemical findings of a patient with primary effusion lymphoma.

**Case presentation:**

A 70-year-old newly diagnosed HIV-positive Ugandan African woman presented with a three-month history of cough, fever, weight loss and drenching night sweats. Three weeks prior to admission she developed right sided chest pain and difficulty in breathing. On examination she had bilateral pleural effusions.

Haematoxylin and eosin stained cytologic sections of the formalin-fixed paraffin-embedded cell block made from the pleural fluid were processed in the Department of Pathology, Makerere University, College of Health Sciences, Kampala, Uganda. Immunohistochemistry was done at the Institute of Haematology and Oncology "L and A Seragnoli", Bologna University School of Medicine, Bologna, Italy, using alkaline phosphatase anti-alkaline phosphatase method. *In situ *hybridization was used for detection of Epstein-Barr virus.

The tumor cells were CD45+, CD30+, CD38+, HHV-8 LANA-1+; but were negative for CD3-, CD20-, CD19-, and CD79a- and EBV RNA+ on *in situ *hybridization. CD138 and Ki-67 were not evaluable. Our patient tested HIV positive and her CD4 cell count was 127/μL.

**Conclusions:**

A definitive diagnosis of primary effusion lymphoma rests on finding a proliferation of large immunoblastic, plasmacytoid and anaplastic cells; HHV-8 in the tumor cells, an immunophenotype that is CD45+, pan B-cell marker negative and lymphocyte activated marker positive. It is essential for clinicians and pathologists to have a high index of suspicion of primary effusion lymphoma when handling HIV positive patients who have effusions without palpable tumor masses. Basic immunohistochemistry is essential for definitive diagnosis.

## Introduction

Primary effusion lymphoma (PEL) is a rare aggressive B-cell lymphoma which was first identified in 1989 as a subset of body-cavity-based lymphomas [1,2]. It accounts for only 0.13% of all AIDS related malignancies among AIDS patients in the USA [3]. The WHO classification recognizes it as a unique entity of non-Hodgkin lymphoma (NHL) [2].

It has been suggested that Kaposi sarcoma herpes virus/Human herpes virus-8 (KSHV/HHV-8) is the causative agent of PEL. In Europe and America very high seroprevalences of HHV-8 (around 67%) have been reported among HIV-positive men who have sex with men [4]. It was initially thought that the transmission in Western countries was sexual [5], however, a recent study in Texas showed high HHV-8 seroprevalences of 26% among children, indicating another mode of transmission of HHV-8 [6].

In Africa, studies have shown considerable variation in the seroprevalence rates of HHV-8 infection among adults and children; the highest adult rates of 26-100% have been found in Uganda, Cameroon, Ivory Coast, Gambia, the Democratic Republic of Congo, Tanzania, Zambia and South Africa.

Nigeria, Ghana, Zimbabwe and Egypt have prevalence rates of 50% and the countries with relatively low rates of 25% and below are the Central African Republic, Eritrea and Senegal [7].

A recent study revealed increasing seroprevalence of HHV-8 with age among Ugandan children, from 10% among two-year-olds to 36.4% in eight-year-olds, in contrast to South African children where there were seroprevalence rates of 7.5-9% [8].

In East and Central Africa, the HHV-8 seroprevalence reaches 80% in the adult population. In Uganda, HHV-8 seroprevalence is approximately 40% in adulthood and studies have also confirmed transmission by blood transfusion [9,10].

In sub-Saharan Africa, there have been very few reports of PEL and this is probably because effusions are often not made into cell blocks, and even when they are, no immunohistochemistry is performed [11,12]. Hence, it is possible that PEL is being missed as an important subtype of AIDS-related NHL [13]. Despite HIV, Epstein-Barr virus (EBV) and HHV-8 being endemic in Uganda, no case of PEL has, before now, been reported from the country. We report a case of PEL in a 70-year-old HIV-infected woman from Kampala, Uganda.

## Case presentation

A 70-year-old newly diagnosed HIV-positive Ugandan African woman presented with a three-month history of cough, fever, weight loss and drenching night sweats. Three weeks prior to admission she developed right sided chest pain and difficulty in breathing. There was no history of haemoptysis or bleeding from any site. She had occasional palpitations. There was no history of leg swelling, orthopnea or paroxysmal nocturnal dyspnoea. She had been vomiting for two days prior to admission, but had no oral sores or alteration in bowel habits. A review of the rest of her systems was unremarkable.

This was her first admission to hospital; she reported having been unwell for three months and had received treatment for cough from a nearby clinic. As she was newly diagnosed with HIV, she had not yet received cotrimoxazole or highly active antiretroviral therapy (HAART).

She was nulliparous, had never married but had had several sexual partners. She had been smoking a pipe (with tobacco); however, she denied alcohol consumption.

On examination, she was elderly, sick looking, with severe pallor, dehydration and wasting. She had no lymphadenopathy, jaundice, or edema. Her liver, spleen and kidneys were not palpable. She had no Kaposi sarcoma lesions on her skin or mucus membranes. She had tachycardia (92 beats per minute) with apparently normal heart sounds but she had a functional systolic murmur. Her blood pressure was 125/90 mmHg. She had tachypnoea (35 breaths per minute), reduced air entry and fine crackles on the right side of her chest.

Results of laboratory investigations are shown in table 1. A chest x-ray showed bilateral pleural effusions and subcutaneous emphysema. Echocardiography showed a mild pericardial effusion.

**Table 1:** Results of laboratory investigations

Laboratory test	Result	Normal range
Serum creatinine	172 μmol/L	44-106 μmol/L

Alanine aminotransferase	8.8 I Units/L	0-41 I Units/L

Serum calcium	2.0 mmol/L	2.2-2.6 mmol/L

Potassium	6.3 mmol/L	3.5-5.5 mmol/L

Sodium	151.2 mmol/L	135-150 mmol/L

WBC (total)	8.5 × 10^3^/μL	4-11 × 10^3^/μL

Neutrophils	62.5%	45-70%

Lymphocytes	22.0%	20-40%

Monocytes	14.4%	3-10%

Platelets	219 × 10^3^/μL	150-400 × 10^3^/μL

Haemoglobin	6.8 g/Dl	12-18 g/dL

CD4 cell count	127/μL	410-1590/μL

CD8 cell count	1178/μL	190-1140/μL

On ultrasonography, her liver, spleen, kidney and pancreas were normal in size, shape and echo pattern. Her urinary bladder wall was normal. There was no ascites or lymphadenopathy. Bilateral large pleural effusions were confirmed. No solid tumor masses were present.

**Figure 1:**
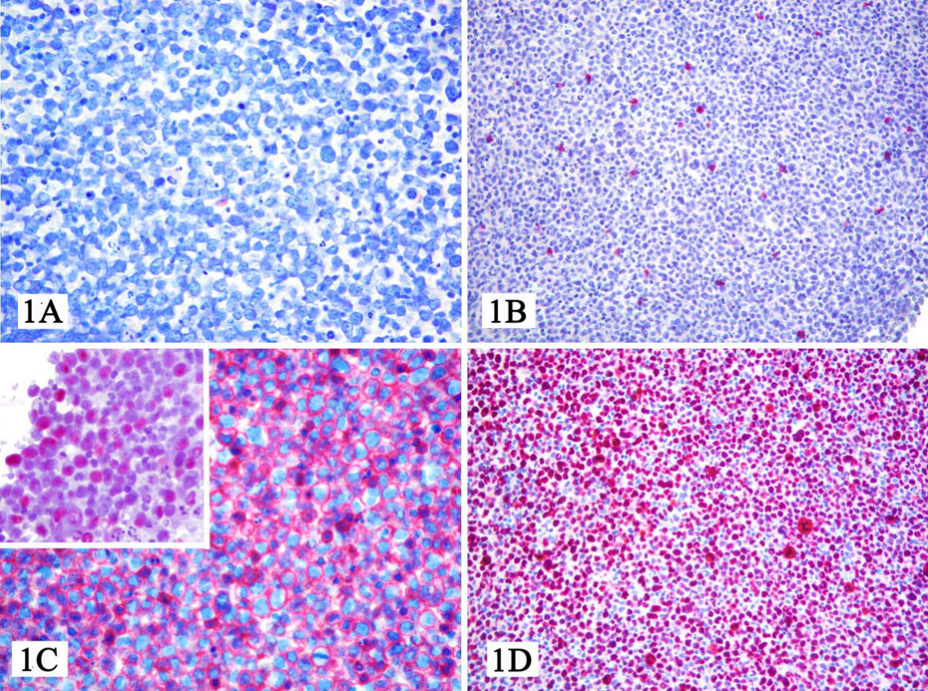
**At light microscopy, the sample consisted of a frankly neoplastic population provided with plasmablastic and/or anaplastic morphology (Figure 1A), which turned out CD3-, CD20- (Figure 1B), CD79a-, CD45+ (Figure 1C), CD38+, CD30+, IRF4+, LANA-1+ (Figure 1D), EBER+ (Figure 1C inset), and Ki-67>90%**. Based on these findings, we made a diagnosis of PEL.

A sample of about 150 ml of pleural fluid was taken from her right hemithorax and sent to the Department of Pathology, Makerere University, College of Health Sciences, Kampala, Uganda, where it was cytocentrifuged. The sediment was made into a cell block and haematoxylin and eosin stained slides revealed a neoplastic proliferation of large lymphoid cells with round to irregular nuclei, prominent nucleoli, and varying amounts of vacuolated cytoplasm. There were immunoblastic, plasmablastic and anaplastic variants with bizarre, pleomorphic nuclei (Figure 1A). They included multinucleated and Reed-Sternberg-like cells. From these findings a preliminary diagnosis of PEL was made.

Since immunohistochemistry is not routinely available in Uganda, it was carried out at the Institute of Haematology and Oncology "L and A Seragnoli", Bologna University School of Medicine, Bologna, Italy. The alkaline phosphatase anti-alkaline phosphatase method and the primary antibodies listed in table 2 were used. *In situ *hybridization was also used for detection of EBV. The tumor cells were CD45+ (Figure 1C), CD30+, CD38+, HHV-8 LANA-1+ (Figure 1D); but were negative for CD3-, CD20- (Figure 1B), CD19-, and CD79a- and EBV RNA+ (Figure 1C inset) on *in situ *hybridization. CD138 and Ki-67 were not evaluable. These findings confirmed the diagnosis of PEL. The patient tested HIV-positive and her CD4 cell count was 127/μL.

**Table 2:** Primary antibodies used for diagnosis

Antibody	Clone	Source	Antigen retrieval	Dilution
CD45	LCA	Dako	EDTA 750 W	1:100

CD3	SP7	Immunotech	EDTA 750 W	1:250

CD20	L26	Dako	EDTA 750 W	1:200

CD30	Ber- H2	Prof. B Falini*	EDTA 900 W	1:3

CD38	SPC32	Novocastra	EDTA 750 W	1:10

CD79a	JCB117	Prof. D Mason^§^	EDTA 750 W	1:10

HHV8	13B10	Menarini	EDTA 750 W	1:10

CD138	-	Neomarkers	EDTA 900 W	1:20

Ki-67	Mib-1	Dako	EDTA 900 W	1:20

*Kindly provided by Prof. Brunangelo Falini, Perugia University, Perugia, Italy.

^§^Kindly provided by Prof. David Y Mason, Oxford University, Oxford, UK.

*Treatment*: She was initially rehydrated with normal saline and 5% dextrose, and later received a blood transfusion. She also received allopurinol, ceftriaxone and metronidazole. After she had stabilized, she was given the first course of chemotherapy with a CHOP protocol (cyclophosphamide 750 mg/m^2^, Adriamycin (doxorubicin) 50 mg/m^2 ^and Oncovin (vincristine) 14 mg/m^2 ^on day one and prednisolone 100 mg on days one to five, repeated every 21 days). In addition, she received omeprazole, metoclopramide and dexamethasone. She registered some improvement after the first course of chemotherapy and was allowed home. She was discharged through the Infectious Disease Institute clinic to be initiated on HAART. She was due to return for subsequent anti-cancer treatment but died two days after discharge from our hospital.

## Discussion

We have reported the case of a patient who presented with all the features of PEL that have been described elsewhere [14,15]. This was the first AIDS defining illness in this patient even though she had very low CD4 counts.

The strong positivity for HHV-8 in this patient is similar to what other authors have found in studies on HIV-positive PEL patients [14,16]. HHV-8, by definition, has to be present in the tumor cells in order to make a diagnosis of PEL [15]. The co-infection of HHV-8 and EBV in this patient is interesting. Both HHV-8 and EBV are γ-herpes viruses and are closely related. While evidence of HHV-8 is essential for diagnosis of PEL, the role of HHV-8 in the pathogenesis is not clear. However, we know that EBV immortalizes B-cells while HHV-8 seems not to. It would, therefore, appear that HHV-8 by itself is not sufficient for the development of PEL. It seems that EBV causes unchecked proliferation of B-cells leading to development of PEL and, according to Fan and others, "once PELs have developed HHV-8 appears to be the driving force [14]." Recently, several cases of EBV negative PEL have been reported implying that HHV-8 plays a critical role in pathogenesis [17].

About 31 cases of HHV-8 independent PEL (HHV-8-unrelated PEL-like lymphoma) have been reported in the literature [18]. This new entity is now referred to more precisely as HHV-8-unrelated large B-cell lymphomas because of the differences observed in its pathogenesis, morphology and immunophenotype (it expresses a B-cell phenotype unlike PEL), and is associated with hepatitis C virus infection in 30-40% of cases [19]. The clinical behavior and prognosis are significantly different from that of PEL patients. The HHV-8-unrelated large B-cell lymphoma patients tend to have more indolent disease that has been observed to resolve spontaneously and, when treated, these patients have a much better prognosis than the HIV-positive, HHV-8 positive PEL patients - who have a very poor outcome [19]. This therefore proves that the two are separate entities.

PEL, possibly uniquely, presents as serous effusions in the pleural, peritoneal and pericardial cavity without identifiable tumor masses or lymphadenopathy.

The morphological presentation is as a large B-cell neoplasm and the tumor cells contain KSHV/HHV-8 DNA and lack c-myc translocations. PEL most commonly affects male AIDS patients and was first recognized in men who have sex with men [20,21]. However, a few cases have been reported in women. It is easily distinguished from other lymphomas because of its unusual morphology with large immunoblastic, plasmacytoid and/or anaplastic cells.

The immunophenotypical presentation of PEL cells is typically as a "null" lymphocyte phenotype, meaning that CD45 is expressed, but routine B-cell (including surface and cytoplasmic immunoglobulin, CD19, CD20, CD79a) and T-cell (CD3, CD4, CD8) markers are absent. Instead, various markers of lymphocyte activation (CD30, CD38, CD71, human leukocyte antigen DR) and plasma cell differentiation (CD138) are usually displayed [22].

PELs are of B-cell origin because they have clonal immunoglobulin gene rearrangements [17,23].

Although immunohistochemical services are not routinely available in Uganda and other resource constrained countries, it is still possible to suspect PEL with available clinical and cytomorphologic criteria. These include lymphomatous effusions limited to the body cavities with no solid tumor or lymphadenopathy and pleomorphic large cells with immunoblastic, plasmacytoid and anaplastic variants [20].

Regarding PEL, no optimal treatment has yet been identified [24]. Most HIV-positive PEL patients receive anthracycline-based multiagent chemotherapy (CHOP; cyclophosphamide, doxorubicin, vincristine, and prednisone) and antiretroviral therapy. Patients with HHV-8 negative large B-cell lymphomas have been shown to benefit from immunotherapy with rituximab since they display B-cell immunophenotype, pleurodesis and thoracocentesis [25]. However, in resource poor settings such as Uganda only CHOP and antiretroviral agents are used [18]. NF-kappa B plays a significant role in PEL oncogenesis. Studies using new drug regimens directed against NF-kappa B have shown positive results. These drugs are currently being developed for therapeutic use [26,27].

## Conclusions

Since a definitive diagnosis of PEL rests on the presence of HHV-8 in the tumor cells, a lymphoproliferation of large immunoblastic, plasmacytoid and/or anaplastic cells, an immunophenotype of leucocyte common antigen CD45 positive, pan B-cell marker negative, and lymphocyte activated marker (CD 138, CD30, CD38, human leukocyte antigen DR and CD71) positive, it is essential for clinicians and pathologists to develop a high index of suspicion of PEL when handling HIV-positive patients with effusions without palpable tumor masses. Basic immunohistochemistry to confirm the diagnosis is necessary.

## Abbreviations

CD: Cluster of differentiation; CHOP: anthracycline-based multiagent chemotherapy (cyclophosphamide, doxorubicin, vincristine, and prednisone); EBER: Epstein-Barr virus encoded RNA; EBV: Epstein-Barr virus; HAART: highly active antiretroviral therapy; HIV: human immunodeficiency virus; HHV-8: human herpesvirus 8; KSHV: Kaposi sarcoma herpes virus; LANA-1: Lymphoma associated nuclear antigen-1; NHL: non-Hodgkin lymphoma; PEL: primary effusion lymphoma; WBC: white blood cell count; WHO: World Health Organization.

## Consent

Written informed consent was obtained from the patient's next-of-kin for publication of this case report and any accompanying images. A copy of the written consent is available for review by the Editor-in-Chief of this journal.

## Competing interests

The authors declare that they have no competing interests.

## Authors' contributions

LKT conceived the idea, made preliminary diagnosis of PEL, and wrote the manuscript. CA carried out immunohistochemistry and *in situ *hybridization. RL, SL, and JO admitted and treated the patient, contributed patient data and revised the manuscript. LOO did cytotechnology and revised the manuscript. PPP and SAP reviewed and revised the manuscript. All authors read and approved the final version of the manuscript.
